# A Pseudouridine Isoxazolidinyl Nucleoside Analogue Structural Analysis: A Morphological Approach

**DOI:** 10.3390/molecules23123381

**Published:** 2018-12-19

**Authors:** Giuseppe Floresta, Venerando Pistarà, Kirsten E. Christensen, Emanuele Amata, Agostino Marrazzo, Davide Gentile, Antonio Rescifina, Francesco Punzo

**Affiliations:** 1 Dipartimento di Scienze del Farmaco, Università di Catania, Viale A. Doria 6, 95125 Catania, Italy; giuseppe.floresta@unict.it (G.F.); vpistara@unict.it (V.P.); eamata@unict.it (E.A.); marrazzo@unict.it (A.M.); davidegentile99@gmail.com (D.G.); 2 Chemical Crystallography, Chemistry Research Laboratory, Mansfield Road, Oxford OX1 3TA, UK; kirsten.christensen@chem.ox.ac.uk

**Keywords:** pseudouridine, pseudouridine 5′-monophosphate glycosidase, isoxazolidine analogs, crystal morphology, polymorphic forms

## Abstract

An in silico study has been conducted upon (3′*RS*,5′*SR*)-5-[2′-benzyl-5′-hydroxymethyl-1′,2′-isoxazolidin-3′-yl]uracil through a molecular dynamics/docking approach that highlights its potential inhibitory activity upon the wild-type pseudouridine 5′-monophosphate glycosidase. The crystal structure of this compound has been solved by means of X-ray single crystal diffraction and the data inferred were used to predict its crystal morphology. These data were compared with optical microscopy images and confirmed the validity of the computed models. This robust approach, already used for several other different compounds, provides a fast and reliable tool to standardize a crystallization method in order to get similar and good quality crystals. As different crystal shapes could be associated with different polymorphic forms, this method could be considered a fast and cheap screening to choose among different and coexistent polymorphic forms. Furthermore, a match with the original crystal structure of pseudouridine 5′-monophosphate is provided.

## 1. Introduction

**Figure 1:**
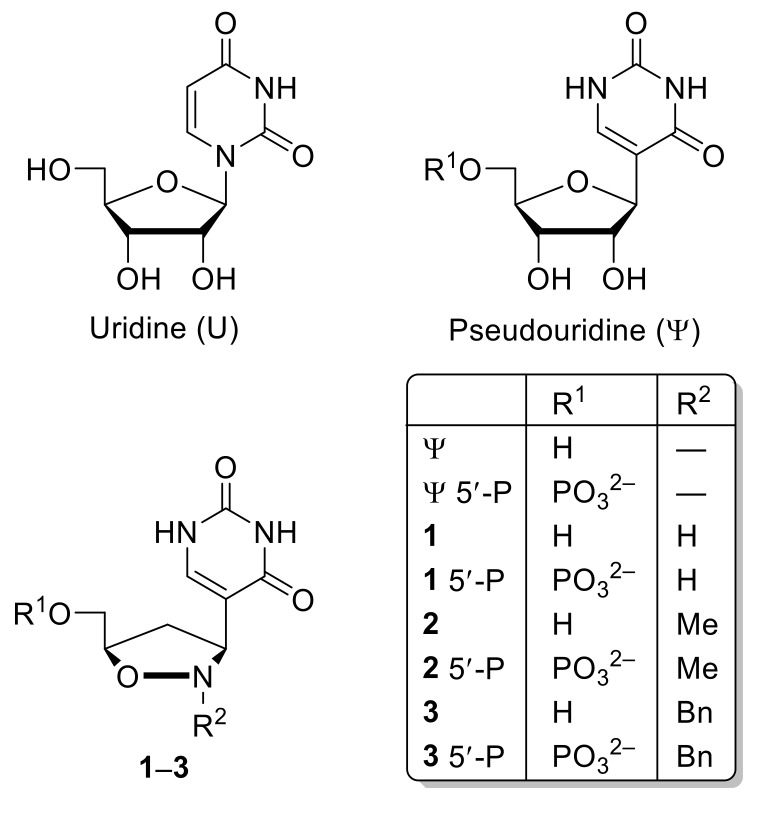
Chemical structures of U, Ψ and of its isoxazolidinyl nucleoside analogs **1**–**3**.

Pseudouridine (Ψ), the *C*-glycoside isomer of uridine (U) (Figure 1) is a ubiquitous constituent of structural RNAs, and it is the most common post-transcriptional modification in all three domains of life (archaea, bacteria, and eukaryote). The enzymes that catalyze the isomerization of U to Ψ are the pseudouridine synthases (Ψ synthases), and they use both sequence and structural information to achieve site-specific generation of Ψ in particular RNA contexts [1].

Pseudouridine is physically and chemically distinct from its parent uridine. The C-C glycosidic bond in Ψ exhibits enhanced rotational freedom as compared to the C-N glycosidic bond in U [2]. Moreover, the C-C glycosidic bond tends to be more stable than the C-N one, and the two nitrogens on the cyclic ring allow for more hydrogen bonding.

CD spectroscopy [3], NMR studies [3], and molecular dynamics simulations [4] suggest that Ψ imparts structural rigidity in both single- and double-stranded RNAs, possibly due to the free imino proton in Ψ that can act as an additional hydrogen bond donor [5]. The presence of Ψ in an *anti*-configuration in polynucleotide chains provides an appropriate geometry for coordination of a water molecule between the imino proton and the 5′-phosphate backbone of its preceding residue, thereby restricting the base conformation and mobility of the backbone [5]. Additionally, Ψ enhances local RNA stacking, which is perhaps the most important contribution of Ψ towards stabilizing the RNA structure [5].

The ubiquity of Ψ across all forms of RNA emphasizes its physiological importance. Mutations in the human gene dyskerin, an ortholog of the Ψ synthase-encoding Cbf5 gene is shown to be associated with the X-linked form of dyskeratosis congenita, a rare hematopoietic and malignant disorder [6]. Individuals with this disorder show increased cancer susceptibility, failure in ribosome biogenesis, and cells with decreased telomerase activity that results in a difficulty in maintaining the telomere length.

Whereas the molecular conformation of U and Ψ, in water solution, have been extensively investigated [7], the corresponding studies in the solid state have received less attention. Although the crystal structures U and Ψ are noted from 1975 [8] and 1997 [9], respectively, only recently the X-ray structure of Ψ 5ʹ-monophosphate glycosidase (Ψ 5′-P Gly) in complex with Ψ 5′-monophosphate (Ψ 5′-P) has been reported, [10] thus lending a new momentum to the interest towards this subject. By performing a modification on both the furanose ring and the heterocyclic base, we achieved a new kind of Ψ based on an isoxazolidine ring (compounds **1**–**3**, Figure 1) [11], whose activity is currently being tested by means of an extensive preliminary in silico screening in our laboratory [12].

From this study, compound **1** proved to be particularly interesting as a potential inhibitor of the Ψ 5′-P Gly [12]. At the same time, compound **3** didn’t get a promising score in our model, probably as a consequence of the presence of different water molecules inside the binding pocket that didn’t allow an easy and favorable accommodation of the benzyl group; in fact, when compound **3** was docked in the pocket without the water consensus molecules, it was as active as Ψ. Since the co-crystallized X-ray structure used by us contained a K166A mutation, necessary to isolate the enzyme with the Ψ substrate in the closed form, we decided to reevaluate the potential activity of molecules **3** using the non-mutated wild-type sequence of Ψ 5′-P Gly (PDB ID: 4GIL) co-crystallized with the ring-opened ribose Ψ 5′-P covalently attached to Lys166, which is even better for the representation of the active protein, according to the mechanism proposed by Huang [10]. Moreover, in this structure, the water molecules inside the binding pocket are absent, as they are displaced by the presence of the co-crystallized ligand. Contemporarily, the aim of this work is offering an in-depth analysis of the solid-state structural features of compound **3**. This analysis is not limited to the atomic level, but extended, by means of the crystal morphology prediction and its correlation with optical microscopy (OM) images, to the microscopic level i.e., the actual crystals, which could be of great interest to a broader scientific audience.

In fact, a detailed crystal morphology analysis will highlight the chemical characteristics of every single grown face, thus enabling an in-depth study of the willingness and ability of each surface to interact with a solvent. As a consequence, a relationship with the compound solubility and its dissolution rate could be inferred, in order to improve it and to explore possible alternative crystallization routes [13]. Finally, we compared the molecular dynamics (MD)/docking results with the crystallographic data.

Since, up to now no molecules have been tested as a Ψ 5′-P Gly inhibitor, and due to the importance of this protein in bacteria as well as to the lack of it in mammals, a new potential class of antibiotics could be born, which could be a path worth pursuing, considering the actual problem of multiple drug resistance (MDR).

## 2. Results and Discussion

### 2.1. In Silico Studies

**Figure 2:**
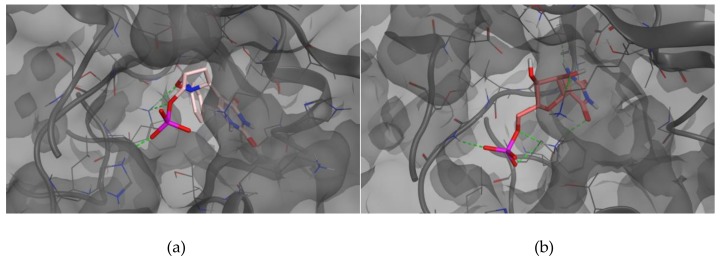
Docked pose of **3** 5′-P inside Ψ 5′-P Gly (**a**) and Docked pose of Ψ inside Ψ 5′-P Gly (**b**).

**Figure 3:**
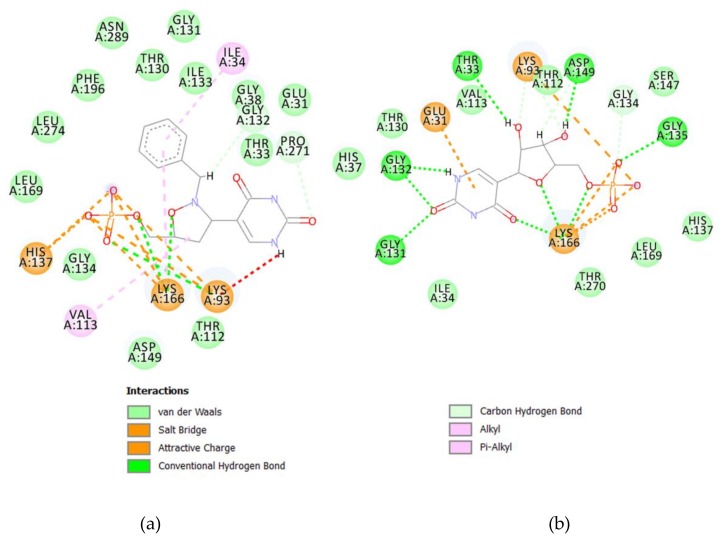
2D molecular interaction of **3** 5′-P (**a**) and Ψ (**b**) with Ψ 5′-P Gly.

To reevaluate Ψ, Ψ 5′-P, **3**, **3**-phosphate, and Ψ-phosphate using the wild-type sequence of the Ψ 5ʹ-P Gly we first docked them into the selected binding sites of Ψ 5′-P Gly and, after the first docking calculation, the best pose was manually selected and the complex (ligand/Ψ 5′-P Gly) was minimized toward a molecular dynamic simulation of 10 ns. Once we finished the MD simulation, each ligand was extracted and re-docked into the binding site. The results are reported in Table 1 and Figure 2 and Figure 3. As shown by the results, molecules **3** are able to be accommodated in the binding site of Ψ 5′-P Gly reaching binding energies better than the native ligand/substrate (Figure 1, Figure 2 and Figure 3).

### 2.2. Crystal Structure Studies

Compound **3** crystallized in the monoclinic C 2/c space group with a = 19.9960(5) Å, b = 16.7246(2) Å, c = 19.1703(5) Å and β = 116.319(3)°. The unit cell has a volume of 5746.5(3) Å^3^, it hosts 16 molecules (Z = 16) with two symmetrically independent fragments (Z’ = 2) as basing building blocks.

The structure shows a complex network of hydrogen bonds (Table 2) both inter- and intramolecular. The whole packing is strengthened by the presence of several *π*-stacking and T-shaped interactions as reported in Table 3 and Table 4. According to the literature [14], the data present in Table 4 could suggest also a difference in “efficiency” among the different reported stacking interactions. In fact, among other criteria, the *π*-stacked rings without any slippage between them are not providing the best environment for an efficient interaction among the generated quadrupoles. For this reason, although the name *π*-interactions may be misleading in this case, T-shaped ones could be favored over parallel ones. The centroid 5/centroid 6 interaction (Cg (5)…Cg (6)) has not slippage reported as the dihedral angles between average planes passing from these rings falls out of the calculation cut-off. The 5-membered ring O2–N1–C5–C4–C3 shows a characteristic envelope conformation (on N1) with pucker parameters [15,16,17,18,19] q_2_ = 0.3831 Å, ɸ_2_ = 42.8048°. As expected, the corresponding ring on the second independent fragment (O32–N31–C35–C34–C33) also shows an envelope conformation (on N31) as well as q_2_ = 0.3838 Å and ɸ_2_ = 221.4092°.

The structure shows a fairly high packing index (70%) [20] and no residual solvent accessible voids. As a consequence, we can assume that **3** is unable to host solvents without modifying its crystal structure. It implies that the attachments of solvent molecules must take place directly from the outer part of the considered faces. This is true whatever a crystallization mechanism is considered, but not in the case of either the classical nucleation theory or the two-step mechanism [21,22].

**Figure 4:**
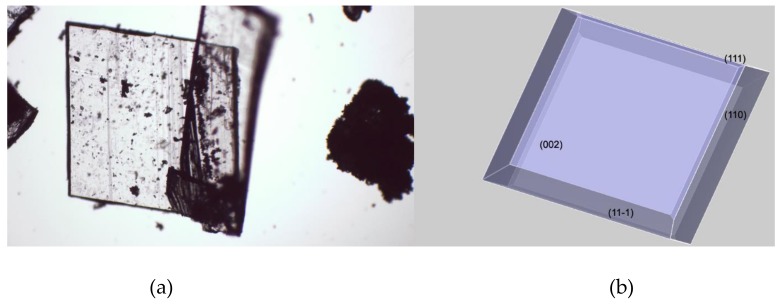
Crystal morphology comparison between the OM collected images (**a**) and the calculated crystal habit (**b**). Miller Indices are reported on the morphologically important (MI) faces.

As shown in Figure 4, there’s an extraordinary matching between the predicted crystal morphology (Figure 4b) and the experimental one (Figure 4a) inferred by means of optical microscopy. It is worth recalling that the crystal morphology prediction is performed in vacuum and therefore it doesn’t take into account any experimental condition such as the role of the solvents used for crystallization. This evident intrinsic limit is substantially overcome if we consider that the angle between crystal faces is controlled by the actual spacing between lattice points—which are characteristic of every considered crystal structure. According to the “law of constancy of interfacial angles” [23], the angles between corresponding faces of the same crystal structure are always the same. This is a consequence of the symmetry of the lattice, which determines the angular relationships between faces. Thus, the experimental conditions that will affect the crystal growth can deviate the expected morphology from its ideal theoretical shape, but can’t change the angles between crystal faces, whether it is a perfect or a distorted crystal.

**Figure 5:**
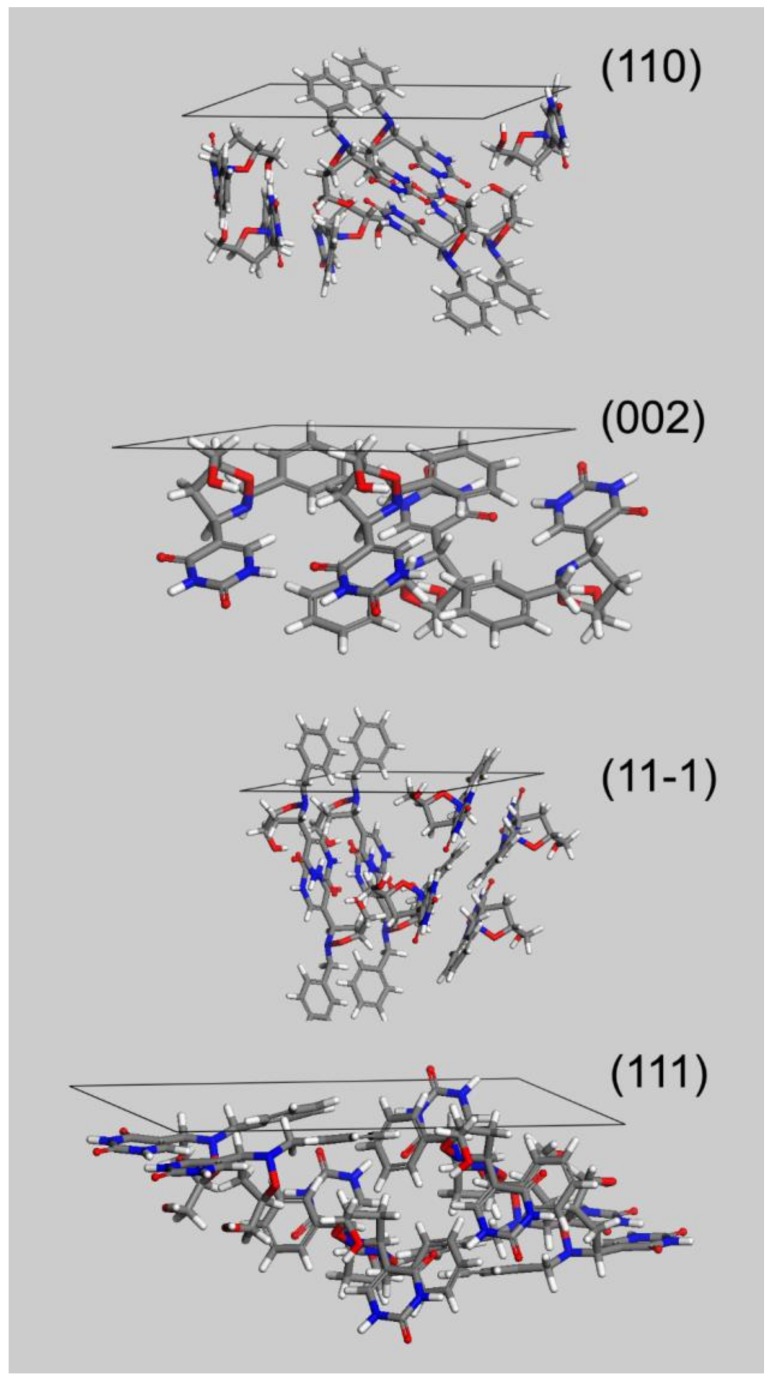
MI faces, and their corresponding Miller indices, for **3**.

The flat and block-shaped crystals show a flat 002 face which represents, in spite of what is the visual inspection, less than the 30% of the whole crystal surface (Table 5). The 110—and symmetry-related—faces instead is by far the most developed ones. The straight analysis of the computed crystal habit doesn’t allow any further consideration than a possible comparison with the experimentally harvested crystals. By analyzing the displacement of the crystal building blocks—i.e., the molecules—just below every single face, it is possible gaining useful information about the interactions of the crystal with different solvents. For this purpose, in Figure 5 we report, for every MI face, the corresponding slab with the relative most protruding molecules just below its surface. This sketch will allow a qualitative analysis, mainly based on a molecule-solvent polarity affinity, which enables further consideration about the opportunity to tune the right solvent choice in order to improve or hinder crystal face growth.

Three of the MI faces show a marked non-polar environment, being (002), (11-1) and (110) all characterized by the prevalence of non-polar groups over strong polar groups. In the case of (002), there is indeed the complete lack of polar synthons. Face (111) on the other hand, while still showing an overall non-polar characteristic, evidently softens this feature as a consequence of the NH-CO-NH sequence on the 6 terms ring. This evidence gives room for speculation about the use of a different solvent mixture to enhance the growth of (111) face or, in turn, to proceed to the detriment of the other MI faces.

Solvent choice, in fact, based on its characteristics such as permittivity and volatility, is undoubtedly the major environmental perturbation potentially occurring during crystallization at room temperature and atmospheric pressure. In a computational morphology prediction, the effect of the solvent is either taken into account directly [24,25,26,27], although with some controversial results, or, as in this work, speculating about the potential solvent effects [25,28,29,30]. While electrostatic interactions among crystal molecules are expected to increase in nonpolar solvents, and decrease in more polar ones due to a shielding effect provided by the solvent itself, solvation/desolvation effects, are expected to show an opposite trend i.e., increase with increasing polarity. This is also the case when the key role in the association forces is played by π-stacking non-covalent interactions [14].

In principle, the use of a non-polar solvent should emphasize the interaction with mostly all the MI face thus slowing the rate of their growth, which corresponds to a better and complete development of the face. In fact, it is useful recalling that the slower the growth process, the bigger and the better the face grows. An opposite situation should be experienced using a less non-polar solvent which should slacken the growth of the less non-polar (111) face thus allowing its better development. The final effect of decreasing solvent non-polarity can result in the growth enhancement of the tiny (111) and symmetry-related pinacoids thus giving rise to a thicker overall habit, such as “block” one by means of the growth of the lateral prismatic sides of the crystal. This is, therefore, another possible morphology that can be expected from the same crystal structure, without performing any dramatic change in the experimental conditions used during crystal growth.

For the above-mentioned reasoning, we can assume that, given the substantial uniformity of the non-polar environment on every considered face, the crystal morphology in presence of a non-polar solvent should be almost independent of the particular solvent used.

Furthermore, we can assume that the reported morphology is characteristic of this particular crystallographic setup i.e., cell parameters and space group [29]. As a consequence, in case of polymorphic structures characterized by a marked difference in cell parameters, or by a different space group in which the molecules crystallize, a fast and visual discrimination can be performed, thus allowing an immediate check of the potential coexistence of different polymorphic forms.

**Figure 6:**
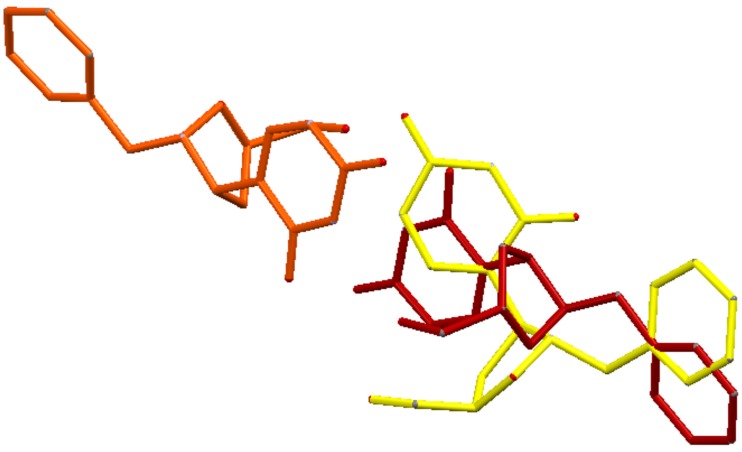
Structures overlay and comparison: X-ray inferred one (red and orange, Z’ = 2) and docked (yellow). (For interpretation of the references to color in this figure legend, the reader is referred to the web version of this article.).

The whole crystallographic characterization allowed us to make a comparison with the molecular layout used to study potential docking features with the pseudouridine 5′-monophosphate glycosidase [12], highlighting a marked difference as reported in Figure 6. As expected, most of the torsional angles in the molecule backbones are significantly changed, thus suggesting a great change from the solid state structure to one which could fit in the molecular pocket of the docking target. In addition, the side rings are evidently tilted to properly fit in the pocket.

**Figure 7:**
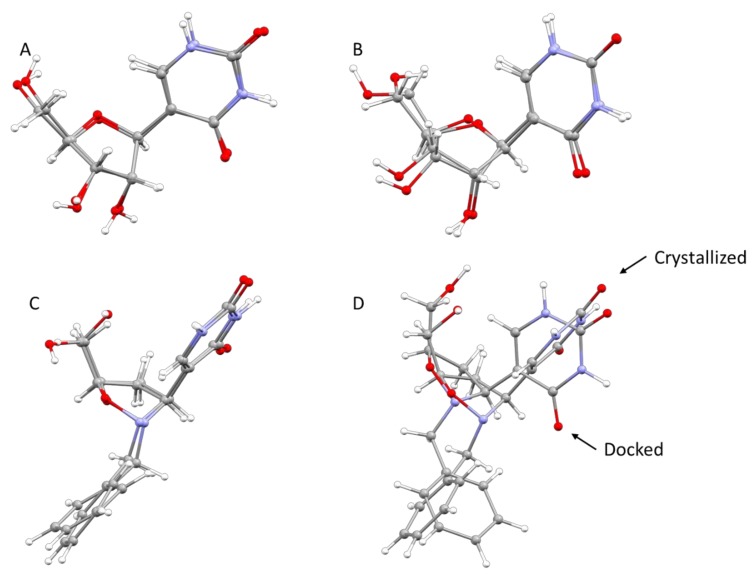
Superposed structures of: (**A**) Ψ from crystal and Ψ from B3LYP/6-31G*; (**B**) Ψ from crystal and Ψ from docking result; (**C**) **3** from crystal and **3** from B3LYP/6-31G*; (**D**) **3** from crystal and **3** from docking result.

As already reported, the proposed mechanism of Ψ cleavage contemplates the acid catalyzed ribose ring opening followed by a covalent linkage between the ribose C1′ and a Ψ 5′-P Gly active site lysine 166 amino group. In light of the mechanism of the Ψ cleavage, the peculiar reactivity of the isoxazolidine ring could prevent the ring-opening process, thus inhibiting the enzyme activity, as postulated by us. The results here obtained clearly demonstrate that even the benzyl analog **3** could act as a potential inhibitor of the Ψ 5′-P Gly. Moreover, another interesting result of our modeling is that we pointed out the structure of Ψ inside Ψ 5′-P Gly, which unfortunately wasn’t obtained previously via X-ray. In fact, the only crystal structure of the enzyme with the Ψ inside the active site was the one with the K166A mutation, and according to the proposed mechanism the lysine residue is of fundamental importance and the mutant form is 2900-fold lower than that of the wild-type. In this way, our obtained “snapshot” of the docked Ψ into the Ψ 5′-P Gly could be used as a starting point for further mechanistic/modeling investigation. The comparison of MD/docking results with the crystallographic data by means of RMSD (Table 6 and Table 7, Figure 7) revealed that basically, no differences between the crystallized and the docked Ψ are present. On the other hand, our compound got a higher RMSD, compared to the one of the Ψ, especially once docked inside the protein, and this was due in major part to the flip of the “uracil” ring and in part to the different accommodation of the benzyl substituent.

## 3. Materials and Methods

### 3.1. Material

Compound **3** was synthesized as previously reported. Briefly, a solution of the appropriate nitrone (5 mmol) and allyl alcohol (5.8 g, 6.8 mL, 100 mmol), in dimethylformamide (DMF) (100 mL), was heated in a sealed tube for 24 h at 140 °C. The solvent was evaporated at reduced pressure and the residue was first purified by flash chromatography column (Merck Group, Darmstadt, Germany) on silica gel (chloroform/methanol, 9:1) and then by a preparative HPLC (Varian PrepStar SD-1, Varian Inc. Palo Alto, CA, USA) [microsorb silica DYNAMAX-100 Å (21 × 250 mm) column, flow 3.5 mL/min] utilizing a mixed isocratic and linear gradient of 2-propanol (10%, 0–15 min, 10–15%, 15–20 min) in *n*-hexane. All spectroscopic data correspond to the literature ones. Compounds **3** was crystallized by dissolving it in a flask with ethanol and allowing a very slow and controlled evaporation of the solvent. Plate, clear and almost colorless crystals were then harvested.

### 3.2. X-ray Single Crystal Diffraction

Data for **3** were collected on Beamline I19 (EH1) [31] at the Diamond Light Source, Didcot, Oxfordshire, where raw frame data were processed (including unit cell refinement, multiscan absorption correction, and inter-frame scaling) using CrystalClear [32]. The structures were solved by Superflip [33], and refined using full-matrix least-squares on F^2^ within the CRYSTALS suite [34]. Non-hydrogen atoms were refined with anisotropic displacement parameters. Further details available in the cif file (see Supplementary Materials).

### 3.3. Optical Microscopy

A trinocular BA310 led Motic microscope equipped with CCIS EF-N Achromat Plan lenses was used for crystals inspection and analysis. A MotiCAM 2500 5.0 MPixel coupled with Motic Image Plus 2.0 ML software (Motic, Hong Kong, China, 2017) was used to collect images.

### 3.4. Crystal Morphology Prediction

The crystal morphology predictions were performed using a preliminary equilibration protocol, by means of the Forcite module included in the Material Studio 7.0 package of Accelrys © (Accelrys, CA, USA, 2013), adopting the molecular mechanics approximation and the Compass II Force Field (FF) [35]. The cif file containing all the crystallographic information was used as the input for an energy minimization, carried out with the Smart Minimizer method. The morphology protocol itself is based on the GM method. For this purpose, the calculations were performed allowing a minimum interplanar distance (d*hkl*) of 0.800 Å without setting any limit neither to the values of the three Miller indices nor to the overall number of growing faces.

The periodic bond chain (PBC) theory by Hartman [36] and Perdock [37,38], is the first methodological attempt to rationalize the influence of the energy related to the interactions between growth units by defining a list of potential grown faces and their ability and tendency to grow. On this basis, it is possible to give rise to the classification of crystal faces by means of the number of PBC belonging to each given adjacent *hkl* layer—a slice—while the crystallization energy is considered a constant for a given crystal. According to literature [29,39,40] there are two main contributions to the overall crystallization energy (*E_cr_*): the energy of each slice (*E_slice_*), i.e., the energy resulting from the lateral interaction of each formula unit within a slice; the attachment energy (*E_att_*) related to the energy released as a consequence of the vertical interaction of the formula unit with an underlying slice [41]. This is summarized as:

*E_cr _*= *E_slice_* + *E_att_* (1)

It was demonstrated [38] that:

*G_r_ ∝ E_att_* (2)

where *G_r_* stands for growth rate. By combining Equations (1) and (2) and by assuming the constancy of *E_cr_*, we get that the bigger *E_slice_*, the smaller *E_att_*, and the slower their *Gr*. A relationship between the molecular interactions—intra- and inter-molecular ones—and the crystal morphology, can be inferred from the combined analysis of both the PBCs present in a slice and the *E_att_* related to it. All these calculations are carried out at 0 K and no surface relaxation takes place. Furthermore, the surface is considered a perfect termination of the bulk.

The search for possible solvent accessible voids was performed using the VOID algorithm [42] setting a grid of 0.20 Å and a probe radius of 1.20 Å.

### 3.5. MD and Docking

All of the compounds were drawn using Marvin Sketch and subjected to a first molecular mechanics energy minimization by Merck molecular force field (MMFF94) optimization using the Marvin Sketch geometrical descriptors plugin [ChemAxon—cheminformatics platforms and desktop applications, v. 18.24, ChemAxon Ltd., Budapest, Hungary, https://www.chemaxon.com/]. The protonation states of the molecules were calculated assuming a pH of 7. After having obtained the 3D structures for all compounds, the geometry was also optimized at semi-empirical level, considering it more precise and effectively successful for the description of small molecules [43,44] and in drug design [45,46,47], using the parameterized model number 3 (PM3) semi-empirical Hamiltonian as implemented in MOPAC package (MOPAC2016 v. 18.151, Stewart Computational Chemistry, Colorado Springs, CO, USA)) [48,49,50]. Docking was performed using AutoDock [51] using the default docking parameters, the point charges were initially assigned according to the AMBER14 force field and then damped to mimic the less polar Gasteiger charges used to optimize the AutoDock scoring function [52]. The setup was done with the YASARA molecular modeling program (v. 18.4.24, YASARA Biosciences GmbH, Vienna, Austria) [53,54,55,56]. The ligands were first docked into the selected binding sites of pseudouridine 5′-monophosphate glycosidase, after the first docking calculation the best pose was manually selected and then the complex (ligand/ pseudouridine 5′-monophosphate glycosidase) was minimized toward a molecular dynamic simulation of 10 ns. Finished the MD simulation each ligand was extracted and re-docked into the binding site. The MD simulation was made in explicit water using YASARA as a software. A 10 Å simulation cell (SC) around all atoms was used. AMBER 14 force field was used for the simulation [57,58]. Simulation temperature was set at 298 K, the SC was uniformly rescaled to reach a pressure of 1 bar, and the pH was set at 7. The simulation was run for 10 ns and single snapshots were recorded every 250 ps.

## 4. Conclusions

In this work, we reconsidered the potential inhibitory activity of molecules **3** using the non-mutated wild-type sequence of Ψ 5′-P Gly, which was proven [10] to be an even better representation of the actually active protein. They proved to be able to establish a strong interaction in the binding site of Ψ 5′-P Gly reaching binding energies better than the native ligand/substrate (Figure 1, Figure 2 and Figure 3). On the basis of these new and formerly [12] undisclosed properties of **3**, we performed a detailed structural analysis of this compound, resolving its crystal structure. The so inferred X-ray data were the starting point for a complete morphological analysis based on the comparison of the experimental data—i.e., real harvested crystals—with in silico predictions. After having checked the correspondence between the predicted crystal morphology and the experimental ones, the effect of solvents of different polarity on the crystal habit has been studied.

Eventually, the evidenced characteristic habit could also be a valuable visual tool to discriminate among different polymorphic forms [59].

As a whole, this study provides additional valuable information about **3,** which will increase overall knowledge about this molecule.

## Figures and Tables

**Table 1:** Calculated binding energies (Δ*G*_bind_) inside the catalytic site of the Ψ 5′-P Gly for all studied compounds.

Compound	Calcd Δ*G*_bind_ (kcal/mol)
Ψ	−6.77
Ψ 5′-P	−7.43
**3**	−8.03
**3** 5′-P	−8.41

**Table 2:** Hydrogen bonding interactions displayed in **3** showing inter- and intramolecular bonds.

Donor-Hydrogen Acceptor	Inter-or Intra-	Donor-Hydrogen (Å)	Hydrogen Acceptor(Å)	Donor-Acceptor	Angle (°)
O7-H71..O15 ^i^	Inter-	0.84	1.97	2.7999(1)	170
N10-H101..O37 ^ii^	Inter-	0.88	1.94	2.8092(1)	171
N12-H121..O44 ^i^	Inter-	0.88	1.83	2.6495(1)	153
O37-H371..O45 ^iii^	Inter-	0.84	1.90	2.7330(1)	169
N40-H401..O7 ^ii^	Inter-	0.86	2.04	2.8949(1)	170
N42-H421..O14 ^iii^	Inter-	0.87	1.91	2.6664(1)	145
C4-H41..O7	Intra-	0.97	2.56	2.9117(1)	101
C13-H131..N1	Intra-	0.95	2.46	2.7852(1)	100
C22-H221..N1	Intra-	0.95	2.56	2.8818(1)	100
C33-H331..O7 ^iv^	Inter-	0.98	2.59	3.5455(1)	165
C34-H342..O15 ^ii^	Inter-	0.96	2.50	3.3793(1)	152
C36-H361..O15 ^v^	Inter-	0.95	2.46	2.7852(1)	100
C43-H431..N31	Intra-	0.93	2.49	2.9043(1)	100
C48-H481..N31	Intra-	0.95	2.57	2.9043(1)	101

Symmetry codes: (i) 1 − x, 2 − y, 1 − z; (ii) x, y, z; (iii) 3/2 − x, 3/2 − y, 1 − z; (iv) x, 2 − y, −1/2 + z; (v) 1 − x, y, 1/2 − z.

**Table 3:** Compact nomenclature for atomic rings (R) and their corresponding center of gravity (Cg) for the aromatic rings in **3**.

Ring #	Center of Gravity #	Atom Numbering
R(1)	Cg(1)	O_2_-N_1_-C_5_-C_4_-C_3_
R(2)	Cg(2)	N_10_-C_9_-C_8_-C_13_-N_12_-C_11_
R(3)	Cg(3)	C_17_-C_18_-C_19_-C_20_-C_21_-C_22_
R(4)	Cg(4)	O_32_-N_31_-C_35_-C_34_-C_33_
R(5)	Cg(5)	N_40_-C_39_-C_38_-C_43_-N_42_-C_41_
R(6)	Cg(6)	C_47_-C_48_-C_49_-C_50_-C_51_-C_52_

**Table 4:** Selected π-π-stacking and T-shaped non-covalent interactions between aromatic rings in **3**. Residues are the collection of Asymmetric Residue Units (ARUs) constituting an isolated unit. Distances between adjacent centroids (Cg) are measured in Å while dihedral angles between average planes passing from each considered ring are measured in (°). Calculations cut-offs were set to Cg–Cg distances <6.0 Å and angles <20° (the latter for π-π only).

Centroid (I)…Centroid (J)	Residue	Distance (Å)	Angle (°)	Slippage (Å)
π-π				
Cg (2)…Cg (2) ^i^	1	5.0610(1)	0	3.943
Cg (2)…Cg (3) ^ii^	1	5.8412(2)	13	4.727
Cg (2)…Cg (5) ^iii^	1	4.5979(1)	4	3.337
Cg (2)…Cg (6) ^iv^	1	4.5182(1)	20	2.906
Cg (3)…Cg (5) ^iv^	1	4.4843(1)	14	2.174
Cg (5)…Cg (2) ^iii^	2	4.5979(1)	4	3.158
Cg (5)…Cg (3) ^v^	2	4.4843(1)	14	2.854
Cg (5)…Cg (5) ^ii^	2	4.9811(1)	0	3.815
Cg (5)…Cg (6) ^vi^	2	5.8581(2)	22	
Cg (2)…Cg (2) ^vii^	2	4.5182(1)	20	2.018
T-shaped				
Cg (5)…Cg (6) ^viii^	2	5.2016(1)	70	
Cg (6)…Cg (3) ^ix^	2	5.2945(1)	78	
Cg (2)…Cg (5) ^x^	2	5.2016(1)	70	

Symmetry codes: (i) 1 − x, 2 − y, 1 − z; (ii) 1/2 − x, 3/2 − y, 1 − z; (iii) x, y, z; (iv) −1/2 + x, −1/2 + y, z; (v) 1/2 + x, 1/2 + y, z; (vi) 3/2 − x, 3/2 − y, 1 − z; (vii) 2 − x, 2 − y, 1 − z; (viii) x,2 − y, 1/2 + z; (ix) 1/2 + x, 3/2 − y, −1/2 + z; (x) x, 2 − y, −1/2 + z.

**Table 5:** Morphology predictions for 1 by means of GM calculations. Percentage of total facet area (TFA) is calculated as 100× (*hkl* facet area)/(total surface area).

*hkl*	Multiplicity	d_hkl_ (Å)	E_att_(Total) (kcal mol^−^^1^)	Total Facet Area (%)
(110)	4	12.2279	−214.9592	57.21
(002)	2	8.5915	−208.9238	27.50
(11-1)	4	11.7887	−299.1849	12.71
(111)	4	8.7861	−255.0837	2.58

**Table 6:** Calculated RMSD of minimized B3LYP/6-31G* compounds from crystal structures.

Compound	RMSD
Ψ	0.18
**3**	0.55

**Table 7:** Calculated RMSD of docked compounds from crystal structures and from minimized B3LYP/6-31G*.

Compound	Crystal Structures	B3LYP/6-31G
Ψ	0.50	0.50
Ψ 5′-P	—	0.94
**3**	1.61	1.75
**3** 5′-P	—	1.65

## Supplementary Materials

The following are available online at http://www.mdpi.com/1420-3049/23/12/3381/s1, CCDC reference number 1882183.

## References

[1] Grosjean H., Benne R. (1998). Modification and Editing of RNA.

[2] Lane B.G., Ofengand J., Gray M.W. (1995). Pseudouridine and O-2’-Methylated Nucleosides—Significance of Their Selective Occurrence in Ribosomal-Rna Domains That Function in Ribosome-Catalyzed Synthesis of the Peptide-Bonds in Proteins. Biochimie.

[3] Davis D.R. (1995). Stabilization of RNA stacking by pseudouridine. Nucl. Acids Res..

[4] Auffinger P., Westhof E. (1998). Effects of pseudouridylation on trna hydration and dynamics: A theoretical approach. Modification and Editing of RNA.

[5] Charette M., Gray M.W. (2000). Pseudouridine in RNA: What, where, how, and why. IUBMB Life.

[6] Heiss N.S., Knight S.W., Vulliamy T.J., Klauck S.M., Wiemann S., Mason P.J., Poustka A., Dokal I. (1998). X-linked dyskeratosis congenita is caused by mutations in a highly conserved gene with putative nucleolar functions. Nat. Genet..

[7] Lipnick R.L., Fissekis J.D., O’Brien J.P. (1981). Structure and conformation of pseudouridine analogues. Biochemistry.

[8] Green E.A., Rosenstein R.D., Shiono R., Abraham D.J., Trus B.L., Marsh R.E. (1975). The crystal structure of uridine. Acta Crystallogr. Sect. B.

[9] Hempel A., Lane B.G., Camerman N. (1997). Pseudouridine. Acta Crystallogr. Sect. C-Cryst. Struct. Commun..

[10] Huang S., Mahanta N., Begley T.P., Ealick S.E. (2012). Pseudouridine monophosphate glycosidase: A new glycosidase mechanism. Biochemistry.

[11] Chiacchio U., Corsaro A., Mates J., Merino P., Piperno A., Rescifina A., Romeo G., Romeo R., Tejero T. (2003). Isoxazolidine analogues of pseudouridine: A new class of modified nucleosides. Tetrahedron.

[12] Floresta G., Pistarà V., Amata E., Dichiara M., Damigella A., Marrazzo A., Prezzavento O., Punzo F., Rescifina A. (2018). Molecular modeling studies of pseudouridine isoxazolidinyl nucleoside analogues as potential inhibitors of the pseudouridine 5′-monophosphate glycosidase. Chem. Biol. Drug Des..

[13] Ibiapino A.L., de Figueiredo L.P., Lima L.M., Barreiro E.J., Punzo F., Ferreira F.F. (2017). Structural and physicochemical characterization of sulfonylhydrazone derivatives designed as hypoglycemic agents. New J. Chem..

[14] Martinez C.R., Iverson B.L. (2012). Rethinking the term “pi-stacking”. Chem. Sci..

[15] Cremer D., Pople J.A. (1975). General definition of ring puckering coordinates. J. Am. Chem. Soc..

[16] Cremer D. (1984). On the correct usage of the Cremer-Pople puckering parameters as quantitative descriptors of ring shapes—A reply to recent criticism by Petit, Dillen and Geise. Acta Crystallogr. Sect. B.

[17] Evans D.G., Boeyens J.C.A. (1989). Conformational-Analysis of Ring Pucker. Acta Crystallogr. Sect. B-Struct. Sci..

[18] Alvarez J.L.G., Amato M.E., Lombardo G.M., Carriedo G.A., Punzo F. (2010). Self-Organization by Chiral Recognition Based on ad hoc Chiral Pockets in Cyclotriphosphazenes with Binaphthoxy and Biphenoxy Substituents: An X-ray, NMR and Computational Study. Eur. J. Inorg. Chem..

[19] Boeyens J.C.A. (1979). The conformation of six-membered rings. J. Cryst. Mol. Struct..

[20] Kitaĭgorodskiĭ A.I. (1973). Molecular Crystals and Molecules.

[21] Vekilov P.G. (2007). What determines the rate of growth of crystals from solution?. Cryst. Growth Des..

[22] Vekilov P.G. (2010). Nucleation. Cryst. Growth Des..

[23] De L’Isle R., Louis J.B. (1783). Cristallographie, ou Description des formes propres a tous les corps du regne minéral, dans l’état de combinaison saline, pierreuse ou métallique.

[24] Chen J., Trout B.L. (2010). Computer-Aided Solvent Selection for Improving the Morphology of Needle-like Crystals: A Case Study of 2,6-Dihydroxybenzoic Acid. Cryst. Growth Des..

[25] Datta S., Grant D.J.W. (2005). Computing the relative nucleation rate of phenylbutazone and sulfamerazine in various solvents. Cryst. Growth Des..

[26] Lee H.-E., Lee T.B., Kim H.-S., Koo K.-K. (2010). Prediction of the Growth Habit of 7-Amino-4,6-dinitrobenzofuroxan Mediated by Cosolvents. Cryst. Growth Des..

[27] Stoica C., Verwer P., Meekes H., van Hoof P.J.C.M., Kaspersen F.M., Vlieg E. (2004). Understanding the effect of a solvent on the crystal habit. Cryst. Growth Des..

[28] Parmar M.M., Khan O., Seton L., Ford J.L. (2007). Polymorph selection with morphology control using solvents. Cryst. Growth Des..

[29] Punzo F. (2011). Space Groups Complexity versus Molecular Interactions in Quinoline Derivatives Crystal Morphology Prediction: A Throughput Evaluation of Different in Silico Approaches. Cryst. Growth Des..

[30] Li Destri G., Marrazzo A., Rescifina A., Punzo F. (2011). How Molecular Interactions Affect Crystal Morphology: The Case of Haloperidol. J. Pharm. Sci..

[31] Nowell H., Barnett S.A., Christensen K.E., Teat S.J., Allan D.R. (2012). I19, the small-molecule single-crystal diffraction beamline at Diamond Light Source. J. Synchrotron Radiat..

[32] (2009). Crystal Clear-SM Expert 2.0 rc14.

[33] Palatinus L., Chapuis G. (2007). SUPERFLIP—A computer program for the solution of crystal structures by charge flipping in arbitrary dimensions. J. Appl. Crystallogr..

[34] Betteridge P.W., Carruthers J.R., Cooper R.I., Prout K., Watkin D.J. (2003). CRYSTALS version 12: Software for guided crystal structure analysis. J. Appl. Crystallogr..

[35] Sun H., Jin Z., Yang C.W., Akkermans R.L.C., Robertson S.H., Spenley N.A., Miller S., Todd S.M. (2016). COMPASS II: Extended coverage for polymer and drug-like molecule databases. J. Mol. Model..

[36] Hartman P. (1980). The attachment energy as a habit controlling factor II. Application to anthracene, tin tetraiodide and orthorhombic sulphur. J. Cryst. Growth.

[37] Hartman P., Bennema P. (1980). The attachment energy as a habit controlling factor: I. Theoretical considerations. J. Cryst. Growth.

[38] Hartman P., Perdok W.G. (1955). On the relations between structure and morphology of crystals. I. Acta Crystallogr..

[39] Docherty R., Clydesdale G., Roberts K.J., Bennema P. (1991). Application of Bravais-Friedel-Donnay-Harker, Attachment Energy and Ising-Models to Predicting and Understanding the Morphology of Molecular-Crystals. J. Phys. D-Appl. Phys..

[40] Punzo F. (2013). Unveiling the role of molecular interactions in crystal morphology prediction. J. Mol. Struct..

[41] Berkovitch-Yellin Z. (1985). Toward an ab initio derivation of crystal morphology. J. Am. Chem. Soc..

[42] Vandersluis P., Spek A.L. (1990). Bypass—An Effective Method for the Refinement of Crystal-Structures Containing Disordered Solvent Regions. Acta Crystallogr. Sect. A.

[43] La Manna P., Talotta C., Floresta G., De Rosa M., Soriente A., Rescifina A., Gaeta C., Neri P. (2018). Mild Friedel-Crafts Reactions inside a Hexameric Resorcinarene Capsule: C-Cl Bond Activation through Hydrogen Bonding to Bridging Water Molecules. Angew Chem. Int. Ed. Engl..

[44] La Manna P., De Rosa M., Talotta C., Gaeta C., Soriente A., Floresta G., Rescifina A., Neri P. (2018). The hexameric resorcinarene capsule as an artificial enzyme: Ruling the regio and stereochemistry of a 1,3-dipolar cycloaddition between nitrones and unsaturated aldehydes. Org. Chem. Front.

[45] Floresta G., Rescifina A., Marrazzo A., Dichiara M., Pistara V., Pittala V., Prezzavento O., Amata E. (2017). Hyphenated 3D-QSAR statistical model-scaffold hopping analysis for the identification of potentially potent and selective sigma-2 receptor ligands. Eur. J. Med. Chem..

[46] Floresta G., Amata E., Dichiara M., Marrazzo A., Salerno L., Romeo G., Prezzavento O., Pittala V., Rescifina A. (2018). Identification of Potentially Potent Heme Oxygenase 1 Inhibitors through 3D-QSAR Coupled to Scaffold-Hopping Analysis. ChemMedChem.

[47] Floresta G., Apirakkan O., Rescifina A., Abbate V. (2018). Discovery of High-Affinity Cannabinoid Receptors Ligands through a 3D-QSAR Ushered by Scaffold-Hopping Analysis. Molecules.

[48] Stewart J.J. (2004). Optimization of parameters for semiempirical methods IV: Extension of MNDO, AM1, and PM3 to more main group elements. J. Mol. Model..

[49] Alemán C., Luque F.J., Orozco M. (1993). Suitability of the PM3-derived molecular electrostatic potentials. J. Comput. Chem..

[50] Stewart J.P. (2016). MOPAC2016.

[51] Morris G.M., Huey R., Lindstrom W., Sanner M.F., Belew R.K., Goodsell D.S., Olson A.J. (2009). AutoDock4 and AutoDockTools4: Automated Docking with Selective Receptor Flexibility. J. Comput. Chem..

[52] Duan Y., Wu C., Chowdhury S., Lee M.C., Xiong G., Zhang W., Yang R., Cieplak P., Luo R., Lee T. (2003). A point-charge force field for molecular mechanics simulations of proteins based on condensed-phase quantum mechanical calculations. J. Comput. Chem..

[53] Krieger E., Vriend G. (2014). YASARA View-molecular graphics for all devices-from smartphones to workstations. Bioinformatics.

[54] Krieger E., Vriend G. (2015). New Ways to Boost Molecular Dynamics Simulations. J. Comput. Chem..

[55] Greish K.F., Salerno L., Al Zahrani R., Amata E., Modica M.N., Romeo G., Marrazzo A., Prezzavento O., Sorrenti V., Rescifina A. (2018). Novel Structural Insight into Inhibitors of Heme Oxygenase-1 (HO-1) by New Imidazole-Based Compounds: Biochemical and In Vitro Anticancer Activity Evaluation. Molecules.

[56] Salerno L., Amata E., Romeo G., Marrazzo A., Prezzavento O., Floresta G., Sorrenti V., Barbagallo I., Rescifina A., Pittala V. (2018). Potholing of the hydrophobic heme oxygenase-1 western region for the search of potent and selective imidazole-based inhibitors. Eur. J. Med. Chem..

[57] Hornak V., Abel R., Okur A., Strockbine B., Roitberg A., Simmerling C. (2006). Comparison of multiple amber force fields and development of improved protein backbone parameters. Proteins Struct. Funct. Bioinforma..

[58] Ponder J.W., Case D.A. (2003). Force fields for protein simulations. Adv. Protein Chem..

[59] Bernstein J. (2002). Polymorphism in Molecular Crystals.

